# Navigating Care Challenges in Elderly Patients Following Hypoglossal Nerve Stimulator Implantation

**DOI:** 10.3390/life15060861

**Published:** 2025-05-27

**Authors:** Michael Joo, Erin Gurski, Efstathia Polychronopoulou, Mukaila Raji, Rizwana Sultana

**Affiliations:** 1 Division of Pulmonary Critical Care & Sleep Medicine, Department of Internal Medicine, University of Texas Medical Branch, Galveston, TX 77555, USA; myjoo@utmb.edu (M.J.); ergurski@utmb.edu (E.G.); 2 Office of Biostatistics, University of Texas Medical Branch, Galveston, TX 77555, USA; efpolych@utmb.edu; 3 Division of Geriatrics, Department of Internal Medicine, University of Texas Medical Branch, Galveston, TX 77555, USA; muraji@utmb.edu

**Keywords:** obstructive sleep apnea, hypoglossal nerve stimulator, elderly, challenges

## Abstract

Introduction: Hypoglossal nerve stimulation (HNS) “Inspire_©_ therapy” has garnered popularity among obstructive sleep apnea (OSA) patients seeking an alternative to continuous positive airway pressure (CPAP) therapy. The growth in HNS has been particularly high in older adults living with OSA. Consistent and proper use of HNS in the geriatric population faces unique age-associated barriers: a high rate of multiple chronic conditions (MCC) and polypharmacy (being on five or more drugs). Early recognition and patient-centered management of these barriers will allow older patients to obtain maximum benefits from HNS. HNS has distinct advantages in the geriatric population because it overcomes many concerns related to CPAP therapy adherence, such as mechanical limitations due to manual dexterity, maxillofacial anatomy, dental issues such as usage of dentures, allergy/otolaryngology-related disorders, and pre-existing post-traumatic stress disorder-related claustrophobia. This paper describes how we worked with older patients with OSA and their care partners to overcome these barriers so patients can continue to derive cardiovascular, neurologic, and quality of life benefits resulting from optimal OSA management. These benefits are especially important in the older population because of higher rates of comorbidities (dementia, coronary artery disease, and atrial fibrillation) exacerbated by sub-optimally treated OSA. In this article, we describe our clinical experience with elderly patients on Inspire_©_ therapy, with a focus on the everyday difficulties faced by these patients and the measures implemented to address and mitigate these barriers. Methods: A retrospective chart review was conducted to identify patients aged 65 and above who underwent hypoglossal nerve stimulator insertion. Experiences of older patients during and after the insertion procedure were documented and compared to a younger population of patients on HNS therapy. We specifically collected information on difficulties encountered during activation or follow-up visits and compared them between the different age groups. Using this information, we identified areas to improve treatment adherence from the patients’ perspectives. Results: We identified 43 geriatric (65 to 86 years old) patients who received the Inspire implant at a tertiary academic medical center and compared them to a younger population of 23 patients. Most common challenges noted—with a potential to impact adherence—included orofacial and lingual neuropraxia (ischemic or demyelination-induced neuropathy) at activation, cognitive dysfunction (memory problems), preexisting anxiety, and insomnia. Other difficulties that are less commonly reported but equally important to consistent and proper use of HNS included headaches, concerns of device malfunction, change in comfort levels after cardiac procedures, and general intolerance of the device. The older patient population had a statistically significant higher incidence of cognitive difficulties (30.2% vs. 4.4%) and a smaller social support system (62.8% vs. 91.3%) affecting device usage compared to the younger population. There were no statistically significant differences in the rates of other more commonly reported adverse effects such as headaches, dry mouth, and anxiety between the two age groups. Conclusion: Despite several challenges faced by geriatric patients, Inspire_©_ hypoglossal nerve stimulation remains a viable, alternative treatment option for OSA with improved tolerance and adherence compared to CPAP. After identifying less commonly reported barriers such as cognitive decline, sensory deficits, and decreased social support systems, minor adjustments and appropriate education on use allows older patients to correctly use and benefit from Inspire_©_ device therapy, with subsequent improvement in sleep and overall quality of life.

## 1. Introduction

Obstructive sleep apnea is one of the most prevalent chronic conditions resulting in significant comorbidities. Globally, it is estimated that nearly 1 billion individuals suffer with obstructive sleep apnea [1,2], with a rising prevalence in the older population [3]. The incidence of this condition has significantly increased with the rising prevalence of obesity [2]. Obstructive sleep apnea is well recognized as a prominent risk factor for multiple cardiovascular and metabolic comorbidities, including hypertension, stroke, coronary artery disease, pulmonary hypertension, atrial fibrillation, diabetes, and depression [2,4].

Various treatment modalities exist for managing sleep apnea, including CPAP (Continuous Positive Airway Pressure), dental advancement devices, weight loss interventions, and surgical procedures [5]. CPAP therapy is considered the gold standard in treatment, demonstrating efficacy even in cases of severe sleep apnea [6]. Nonetheless, adherence poses a significant challenge—especially in the elderly population, leading to non-compliance, inadequate management of sleep apnea, and suboptimal outcomes. Research indicates that long-term CPAP adherence ranges from 40 to 60% of OSA patients [6,7], with a lower rate in the elderly population, a reflection of multiple factors, including age-related increases in cognitive and sensory impairment, polypharmacy, and multimorbidity.

Consequently, there has been a greater interest in alternative surgical interventions, such as the hypoglossal nerve stimulator. This approach has garnered popularity among patients seeking an alternative to CPAP therapy, exhibiting both efficacy and safety [8,9]. Nonetheless, reports highlight multiple potential adverse events, ranging from minor complications to rare but serious occurrences like pneumothorax, vascular injury, device extrusion, malfunction, and migration [10,11,12,13,14].

Despite the growing acceptance of hypoglossal nerve stimulation (HNS), there remains a lack of data concerning the common and potentially modifiable barriers to HNS adherence encountered by patients, particularly among the elderly. While there are shared barriers or adverse events irrespective of age, such as oral or lingual pain, dry mouth, and peri-oral weakness, as highlighted in studies by Doug Chieffe et al. in pediatric patients and Mihai Bentan et al. in the general adult population [15,16], the combination of learning to use new technologies with age-related cognitive decline and comorbidities creates additional barriers for the elderly. Unlike other implantable devices such as pacemakers, pulmonary artery pressure monitors, and pain pumps that are intermittently manipulated primarily by physicians, using HNS daily requires a high degree of familiarity with HNS operating instructions by both the patients and their care providers. Since FDA approval for the treatment of OSA in 2014, there are currently no studies—to our knowledge—specifically addressing the perspectives of patients aged 65 and older, with regard to difficulties and challenges they face when using HNS therapy. The existing literature primarily focuses on adverse events as reported by the Manufacturer and User Facility Device Experience (MAUDE) database, such as pain and device complications like infection or migration. Less threatening but equally serious barriers to consistent and proper use of HNS, such as cognitive and psychological barriers, remain underreported. Thus, it is essential for physicians to identify and understand these patient barriers to provide optimal OSA care for the older population. Drawing from our experience with this population, we have identified unique hurdles and potentially modifiable barriers specific to this age group. In this article, we delve into the everyday barriers faced by elderly patients undergoing hypoglossal nerve stimulation and discuss measures to address them.

## 2. Materials and Methods

A retrospective chart review was conducted to identify patients aged 65 and older who underwent hypoglossal nerve stimulator insertion at a tertiary academic medical center for the treatment of sleep apnea. Patients were divided into two groups: those aged 65 and older, and those younger than 65. We documented the experiences of patients during and after the insertion procedure as well as the difficulties encountered during activation and follow-up visits. Additionally, we gathered insights on areas for improvement from the patients’ perspectives and the subsequent management of identified barriers by patients and their providers via chart review.

Inclusion criteria comprised individuals who were diagnosed with obstructive sleep apnea, underwent hypoglossal nerve stimulator insertion, and had at least one follow-up clinic visit after activation. Patient-reported barriers, adverse events, and their management were recorded in clinic notes.

Exclusion criteria included patients with a diagnosis of obstructive sleep apnea who underwent hypoglossal nerve stimulator insertion but did not have a follow-up visit.

We conducted statistical analysis comparing data between elderly and younger patients to determine whether elderly individuals experience a higher incidence of problems.

## 3. Results

Table 1 shows our sample population patient characteristics and frequency of identified challenges stratified by age group: age ≥ 65 versus age < 65).

We identified 43 elderly patients who received the Inspire implant at a tertiary academic medical center through medical charts review. This group consisted of 23 females and 20 males. We compared this patient cohort to a younger cohort of 23 patients, including seven females and 16 males, to assess whether elderly individuals experience a higher incidence of complications.

Other common difficulties encountered included orofacial and lingual neuropraxia (ischemia or demyelination-induced neuropathy) at activation, cognitive dysfunction (memory problems). complaints of device not working later in the night, change in comfort levels after cardiac procedures, and general intolerance of device. By working with patients and their care partners, we used both cognitive behavioral therapy for insomnia (CBT-I) and pharmacological interventions (acetaminophen analgesic) to mitigate these difficulties—leading to consistency in HNS therapy usage.

## 4. Discussion

In this study, we described and compared the perspectives of patients aged 65 and older to those younger than 65. With the goal of informing clinicians how to work with elderly patients and their care partners to overcome challenges to HNS therapy, below are barriers we identified and our approaches to mitigating them, including a discussion of how our experiences and observations compared with findings from the existing literature [14,15,16,17,18,19,20,21].

1. Cognitive dysfunction and sensory problems

The most common barrier faced by the elderly population is cognitive/sensory dysfunction, which poses unique challenges when using hypoglossal nerve stimulators. Age-related cognitive decline, pre-existing cognitive impairments, and sensory deficits such as impaired vision and hearing can complicate the operation and maintenance of such devices, requiring users to follow up more frequently in the clinic for re-education and reassessment of subjective benefits. These cognitive difficulties may lead to improper usage, inconsistent therapy, and increased frustration due to unmet expectations. Additionally, older adults are more likely to have multiple comorbid conditions, complicating device usage and clouding the subjective description of treatment benefit. Patients and their spouses or bed partners may also experience hearing loss, making it difficult to report snoring or witnessed apneas. Hence, intensive involvement of a patient’s care partners as well as comprehensive support and education tailored to these populations are crucial for the effective utilization of the Inspire system.

Another challenge faced was patients’ unfamiliarity with technology like smartphones [20]. Patients with cognitive impairments or dementia found it hard to use the remote control and connect it to their phones. They struggled with basic functions, including adjusting HNS settings and linking the device to smartphone applications. Overcoming these challenges often requires multiple clinic visits by patients and their care partners before becoming comfortable and adept at operating the HNS. Those with memory loss often needed assistance from family members during clinic visits. In some cases, home health visits—by a multidisciplinary team of healthcare professionals such as occupational therapists or physicians—may be needed to ensure safety and assess home situations; such visits have been shown to be beneficial in older patients transitioning from the hospital to home with medical devices to ensure safe usage [17]. We address these difficulties with the active participation of patients and their families/caregivers in all phases of the procedure and thereafter.

Despite receiving thorough instructions, certain patients persisted in utilizing the lowest settings for an extended duration, forgetting how to adjust treatment levels. Surprisingly, they still reported improved well-being, possibly indicative of the placebo effect at play. To facilitate their adjustment, we recommended that patients consider bringing a family member or care partner along if feasible to learn and aid in operating the remote until they felt self-confident.

Impaired cognition can also lead to HNS intolerance. While intolerance can be caused by various factors, one straightforward reason was that patients were rapidly adjusting the levels without realizing it. We discovered that these patients were placing the remote directly on their chest and pressing buttons without directly looking at the remote. This unintentionally caused them to inadvertently change levels, going up one or multiple levels at a time, resulting in complaints of intolerance. We now educate every patient on the importance of holding the remote in their hands and visually confirming which buttons they are pressing before placing the remote near their implant.

Activation visits tend to be lengthier for elderly patients compared to their younger counterparts due to the above-mentioned problems. To manage this difficulty, we used a comprehensive education approach that utilized technology, such as connecting to a smartphone application for remote compliance reporting, remembering login credentials, learning how to use the remote, etc., necessitating extended time for education and re-education during the activation visit.

2. Anxiety and Insomnia

Patients with anxiety and insomnia often have heightened sensory responses to initial stimulation along with anxious anticipation about when the device will activate. Anxiety and insomnia were equally prevalent in both age groups. The coexistence of insomnia and anxiety contributes to poor sleep hygiene, an erratic sleep schedule, and unpredictable social factors that impact compliance [21]. Excessive worry and fear significantly affect the user’s experience, hindering their ability to fall asleep within the designated start delay time. Persistent rumination on the impending stimulation makes it challenging for patients to both initially fall asleep and return to sleep upon waking in the night and feeling the stimulation. Similar findings of worsening psychiatric comorbidities have been reported in patients with atrial fibrillation and wearable cardiac monitors, with up to 20% of patients reporting intense fear, preoccupation, and anxiety with device notifications [18].

These patients benefit from regular follow-up appointments to adjust settings such as pulse/width, amplitude, and extending start delay or pause times. Modifying electrode settings and maintaining a low amplitude with gradual titration often proves beneficial. In some cases, the addition of a mild sedative like low-dose Trazodone for a short period can aid in the initial adjustment phase. Follow-ups included focused discussions on sleep hygiene techniques, and medication adjustments were proven helpful.

3. Headaches

Although not statistically significant, four patients in the elderly group experienced either new headaches or a worsening of their existing headaches following surgery as compared to one patient in the younger group. We delayed activation and ended up activating after 3-4 months post-surgery. Their headaches intensified as we increased the voltage. We had to adjust the electrode configuration to the weakest electrode configuration (E) and alter the pulse/width. These patients needed a very gradual increase in their amplitude, spanning over a period of months. Sometimes, we advised the patient to take low-dose acetaminophen 650 mg at bedtime, as needed. Though not as common as pain at the device and lead sites, pain in the head/face region was noted in 6.1% of the patients listing pain as an adverse event in a MAUDE database review [19].

4. Dry mouth

Both groups exhibited signs of dry mouth. Hypoglossal nerve stimulation, which moves the tongue forward, may exacerbate dry mouth in this population, leading to heightened discomfort especially if the patient is a mouth breather. Though this was not found as an adverse effect in the MAUDE study [19], it was reported as an issue in Facebook groups [16]. This likely reflects reporting bias, as the MAUDE database primarily reflects serious, adverse effects that would motivate patients to voluntarily report. The elderly population can experience dry mouth due to various factors, including medication side effects and systemic diseases such as type II diabetes and Alzheimer’s disease, as well as dehydration. Complete resolution of dry mouth is challenging, requiring more frequent follow-ups post-activation as well as safe de-prescribing of anticholinergic medications. Potential solutions involve modifying the amplitude range or incorporating additional adjunct therapies such as using a chin strap, a humidifier, or mouth rinses that coat the oral cavity and tongue. Collaborating with the primary care physician to discuss medication adjustments can be a beneficial option.

Other common observations:

1. Post-surgery activation timeline

Inspire, the manufacturer of the Hypoglossal nerve stimulator, recommends a delay of 4 weeks post-implant surgery before device activation. This period allows for essential healing, ensuring optimal results and patient well-being. We have observed a trend among the elderly population post-surgery, with frequent cases of neuropraxia consistent with findings from other studies analyzing the MAUDE database [10,19] This has necessitated postponing activation, leading to patient dissatisfaction and a strain on clinic resources, including the dedicated hour allocated for Inspire activation visits. Among our initial 14 patients, five required delayed activation, underscoring the significance of this issue. Additionally, we have noted elevated sensational and functional thresholds, further highlighting the importance of careful consideration and management in these cases.

To manage this difficulty, we have revised our approach by extending the post-surgery activation timeline to 6 weeks. This adjustment allowed for ample healing time, resulting in a notable absence of neuropraxia (peripheral neuropathy related to ischemia or demyelination) among patients at the time of activation. Consequently, there were fewer instances of rescheduled appointments, and patients experienced greater satisfaction, as their expectations aligned with the revised schedule.

2. Feeling of inadequate treatment

Many elderly patients commonly reported feeling like the device was not working, especially later in the night. This feeling can be attributed to adjustments in the tongue muscles to the device’s amplitude. Some patients panicked, leading to a quick increase in amplitude levels in the device setting and subsequent device intolerance from overstimulation or stimulation discomfort, findings consistent with reports from past studies [16,19]. This discomfort resulted in frequent requests for early follow-up appointments and in-clinic adjustments. Reassurance was provided, and the device was checked again. Patients were reminded to ensure that the rim of the remote is solid green, indicating proper functioning, and the importance of gradually adjusting the amplitude was emphasized.

3. Change in comfort levels after atrial fibrillation ablation

Two patients underwent ablation for newly diagnosed atrial fibrillation. They reported feeling that HNS intensity was higher and were not able to tolerate the same amplitude prior to their ablation. On examination, tongue protrusion was acceptable, but voltage was lowered to subtherapeutic levels to improve tolerance. These patients required slow re-titration to reach the therapeutic voltage levels again. Though there is no similar scenario documented in the current literature, this phenomenon may be lumped under the umbrella category of stimulation discomfort, which is more commonly reported in Facebook group posts as opposed to the MAUDE database [16]. As the elderly population is more prone to developing cardiac comorbidities such as arrhythmias and more likely to undergo such procedures, it is especially important to assess their tolerance and comfort after such procedures.

## 5. Conclusions

Hypoglossal nerve stimulation therapy is gaining rapid popularity among obstructive sleep apnea patients who struggle with CPAP therapy intolerance. Despite the difficulties encountered by elderly patients on Inspire therapy, this treatment option holds significant promise, contingent upon diligent guidance and the active involvement of family members until patients establish their optimal settings. With minor adjustments and targeted re-education, these patients successfully integrate the device into their routines, often reporting subjective improvements in their therapy outcomes.

## Figures and Tables

**Table 1:** Description of patients with obstructive sleep apnea and frequency of challenges associated with Inspire therapy, stratified by age group: age ≥ 65 versus age < 65.

	Older Group, ≥65 N = 43	Younger Group, <65 N = 23	*p*-Value
**Gender, N (%)**			0.0731
Females	23 (53.5)	7 (30.4)	
Males	20 (46.5)	16 (69.6)	
**Social support, N (%)**			0.0190
No	16 (37.2)	2 (8.7)	
Yes	27 (62.8)	21 (91.3)	
**Any adverse effects, N (%)**			0.4227
No	18 (48.9)	12 (52.2)	
Yes	25 (58.1)	11 (47.8)	
**Anxiety/Insomnia, N (%)**			0.7596
No	34 (79.1)	17 (73.9)	
Yes	9 (20.9)	6 (26.1)	
**Cognitive difficulty, N (%)**			0.0241
No	30 (69.8)	22 (95.6)	
Yes	13 (30.2)	1 (4.4)	
**Dry mouth, N (%)**			1.0000
No	38 (88.4)	20 (87.0)	
Yes	5 (11.6)	3 (13.0)	
**Connection difficulties, N (%)**			0.5461
No	40 (93.0)	23 (100)	
Yes	3 (7.0)	0 (0)	
**Headache, N (%)**			0.6506
No	39 (90.7)	22 (95.7)	
Yes	4 (9.3)	1 (4.4)	

Comment: Overall mean age = 66 years (SD = 10.5). The older group has statistically significantly less social support (caregiver or spouse) than the younger group (62.8% vs. 91.3%) and the older group has significantly more cognitive difficulties (30.2% vs. 4.4%). Patient age ranged from 65 to 86 years old, with mean age = 66 years (SD = 10.5). We compared the presence of any difficulties, social support system, anxiety/insomnia, cognitive issues, dry mouth, headaches, and challenges using the remote between the two groups. The older group reported significantly less social support (caregiver or spouse) compared to the younger group (62.8% vs. 91.3%) and experienced more cognitive difficulties (30.2% vs. 4.4%). There was no statistically significant difference between the two groups in terms of insomnia, anxiety, dry mouth, or headaches.

## Data Availability

The original contributions presented in the study are included in the article, further inquiries can be directed to the corresponding author.
